# Global burden of early-onset pancreatic cancer attributable to metabolic risks from 1990 to 2021, and projections to 2030

**DOI:** 10.3389/fonc.2025.1660313

**Published:** 2025-10-15

**Authors:** Yingjin Fang, Yile Xu, Faliang Xing, Weixin Zhang, Chen Liang, Qingcai Meng, Jialin Li, Jin Xu, Wei Wang, Yi Qin, Xianjun Yu, Bo Zhang

**Affiliations:** ^1^ Department of Pancreatic Surgery, Fudan University Shanghai Cancer Center, Shanghai, China; ^2^ Department of Oncology, Shanghai Medical College, Fudan University, Shanghai, China; ^3^ Shanghai Pancreatic Cancer Institute, Shanghai, China; ^4^ Shanghai Key Laboratory of Precision Medicine for Pancreatic Cancer, Shanghai, China; ^5^ Pancreatic Cancer Institute, Fudan University, Shanghai, China; ^6^ Department of Pediatric Surgery, Guangzhou Institute of Pediatrics, Guangdong Provincial Key Laboratory of Research in Structural Birth Defect Disease, Guangzhou Women and Children’s Medical Center, Guangzhou Medical University, Guangdong Provincial Clinical Research Center for Child Health, Guangzhou, Guangdong, China

**Keywords:** early-onset pancreatic cancer, disease burden, age-standardized mortality rate, age-standardized disability rate, Bayesian age-period-cohort

## Abstract

This study, based on the Global Burden of Disease (GBD) 2021 data, systematically analyzed the changes in the disease burden of early-onset pancreatic cancer (EOPC) attributable to high fasting plasma glucose (HFPG) and high body mass index (HBMI) among the global population aged 15–49 years from 1990 to 2021 and predicted the mortality trends up to 2030. The results show that metabolic risk factors have a significant impact on EOPC: In 2021, the global deaths from EOPC attributable to HFPG reached 3,334 cases, 2.3 times higher than in 1990 with the age-standardized mortality rate (ASMR) and age-standardized disability rate (ASDR) had average annual growth rates of 1.50% and 1.47%. The ASMR and ASDR growth rates attributable to HBMI were even higher (1.69% and 1.76%). The steepest ASMR increases occurred in low-middle socio-demographic index (SDI) regions with an average annual growth of 2.86%), while the highest absolute burdens were observed in East Asia, high-income North America, and Western Europe. Bayesian age-period-cohort (BAPC) model predictions indicate that by 2030, the ASMR related to HBMI will continue to rise in both sexes (from 0.90 to 1.65 per 100,000 in males and from 1.43 to 1.93 per 100,000 in females), and the HBMI may exert a greater impact on females than HFPG. The study reveals the “double burden” phenomenon of metabolic risks: high-SDI regions have a high absolute burden due to the accumulation of long-term metabolic diseases, while low-middle SDI regions experience significant growth rates due to rapid urbanization and a lack of medical resources. Gender difference analysis shows that males generally have a higher ASMR than females, but the upward trend of metabolic-related mortality rates in females is more severe. The interaction between behavioral pattern changes in young people and metabolic abnormalities further exacerbates the risk. This study provides temporospatial evidence for the prevention and control of global EOPC, emphasizing the need to strengthen interventions for metabolic diseases in middle-and low-income regions, optimize the allocation of medical resources, and prioritize gender- and youth-specific interventions to curb the global spread of this aggressive cancer.

## 1 Introduction

Pancreatic cancer (PC) is a highly aggressive neoplasm of the digestive system that poses a major threat to public health. Early-onset PC (EOPC) is generally defined as PC diagnosed in individuals younger than 50 years of age (1) and has received increasing attention in recent years. Over time, the global incidence of EOPC has shown a significant upward trend. A study analyzing data from the Surveillance, Epidemiology, and End Results (SEER) program found that between 1992 and 2015, the incidence of PC in individuals aged< 50 years increased at an annual rate of approximately 1.5% (2). The age-standardized rates (ASRs) of incidence, mortality, and disability-adjusted life years (DALYs) for EOPC in Africa, the Americas, and Asia are continuously increasing (3). Patients with EOPC often have poorer prognoses than patients with late-onset PC because they are frequently diagnosed at advanced stages, experience shorter survival times, and have a lower quality of life (4).

With the escalating global incidence and poor prognosis of EOPC, identifying its potential risk factors and formulating targeted prevention and intervention strategies have become urgent public health priorities. Among the various factors under investigation, metabolic-related conditions have emerged as critical modifiable contributors to EOPC risk, with high body mass index (HBMI) and high fasting plasma glucose (HFPG) being identified as two well-validated risk factors (5).

Studies have consistently confirmed that HFPG is strongly correlated with an increased PC risk. Under hyperglycemia, insulin resistance elevates insulin levels and activates intracellular signaling pathways that promote PC cell proliferation, survival, and metastasis (6). Elevated insulin enhances insulin-like growth factor-1 bioavailability to drive cellular growth (7). Additionally, HFPG induces oxidative stress and inflammatory responses, damages the pancreatic tissue, and increases PC risk (6). Meta-analyses have shown that diabetic individuals have a two-to three-fold higher PC risk than non-diabetic individuals, with type 2 diabetes being more closely associated and long-term HFPG and insulin resistance being key contributing factors (8, 9). Population-based studies across regions and age subgroups validate this finding based on quantitative data. A Swedish nested case-control study found that each 1 mmol/L increase in fasting glucose levels 5–15 years before diagnosis increased the risk of pancreatic ductal adenocarcinoma (PDAC, the most common subtype of PC) by 14% (10). A UK Biobank analysis (patient age 40–54 years) showed that HbA1c ≥ 6.5% was associated with a 2.1-fold higher risk of early-onset PDAC (11). UK CPRD data noted that persistent hyperglycemia 3 years before diagnosis increased PDAC risk 2.7-fold in those aged<55 years (12), whereas another study reported that impaired fasting glucose in 20–44-year-olds showed a hazard ratio (HR) of 1.73 (13). Further, a systematic review reported that each 1 mmol/L increase in fasting glucose correlated with a 14% higher PDAC risk in those aged<60 years (14), and the US Cancer Prevention Study-II found that diabetic patients aged<55 years had a relative risk (RR) of 2.3 for PC mortality (15).

HBMI, which is an indicator of overweightness and obesity, is a significant risk factor for EOPC. Obesity is a chronic inflammatory condition in which the adipose tissue releases a substantial quantity of inflammatory mediators and adipokines that can disrupt the normal functioning of pancreatic cells and facilitate PC onset and progression (16, 17). A longitudinal study of 1.79 million adolescents confirmed that adolescent obesity is associated with an increased risk of adult PC (18), with the association being stronger when a continued increase in BMI is associated with a steeper increase in risk. Cumulative epidemiological evidence from multicenter studies and pooled analyses clarifies the dose-response and age-specific impact of BMI on EOPC risk; a US multicenter case-control study found that obese individuals (BMI ≥ 30 kg/m²) aged 18–21 years had an odds ratio (OR) of 1.9 for early-onset PDAC, with a mean diagnosis age 2–6 years earlier (19). The PanScan Consortium (10 pooled cohorts) found that for every 5 kg/m² BMI increase, the HR for EOPC was 1.13 in those aged<55 years (20). Moreover, a pooled analysis of 14 cohorts confirmed that 18–21-year-old overweight/obese individuals had an RR of 1.2 for EOPC, and adults with >10 kg/m² increase in BMI had an RR of 1.4 (21). An earlier pooled study reported that patients aged<60 years with BMI ≥ 30 kg/m² had an RR of 1.9 for PDAC (22), while 50–59-year-old obese individuals in the NIH-AARP cohort had an HR of 1.47 for PDAC (23).

Disease burden is a comprehensive indicator used to describe the impact of specific diseases or disease categories on population health and socioeconomic factors. By studying the global disease burden of EOPC caused by HFPG and HBMI, we can gain a clear understanding of the incidence of EOPC in different regions and populations, and the roles that HFPG and HBMI play in this context. This can help us identify high-incidence and high-risk populations, providing a basis for formulating precise prevention strategies. By predicting the future disease burden of EOPC, we allocate medical resources in advance, strengthen the screening and monitoring of high-risk populations, improve early diagnosis rates, and enhance patient prognosis. In-depth research of the relationship among HFPG, HBMI, and EOPC can also provide a theoretical foundation for developing new therapeutic targets and drugs, thereby promoting advancements in PC treatment.

Currently, there is relatively little research on the global disease burden of EOPC caused by HFPG and HBMI, and predictive data on the future disease burdens are lacking. Given the alterations in global lifestyle and the escalating incidence of obesity and diabetes, the burden of EOPC is expected to increase. Therefore, this study aimed to systematically examine the global disease burden associated with HFPG levels and HBMI. Additionally, it aimed to predict the disease burden for the year 2030, thereby offering an evidence base for the prevention, management, and treatment of EOPC on a global scale.

## 2 Methods

### 2.1 Data source

We compiled data from the 2021 Global Burden of Disease database (GBD 2021), including annual deaths, DALY counts, age-standardized mortality rates (ASMRs), and age-standardized DALY rates (ASDRs) for EOPC attributable to HFPG and HBMI. The analysis included global data, five sociodemographic index (SDI) regions, and 21 GBD-defined regions from 1990 to 2021. Data were sourced from the Global Health Data Exchange (24).

### 2.2 Definitions and measures

EOPC, denoting cases confirmed in patients aged 15–49 years, was classified using the application of the International Classification of Diseases, 10th Revision diagnostic codes C25-C25.9 (24). These codes were used in previous studies (25, 26). Death cases and DALYs counts were estimated with 95% uncertainty intervals (UIs) based on 1000 posterior draws, represented by the 2.5th and 97.5th ranked values. Age-adjusted metrics were computed using a direct approach based on demographic projections from the GBD 2021 standard population. Analytical procedures for estimating age-standardized metrics have been described in previous studies (27). In this study, we identified HFPG and HBMI as metabolic risk factors for EOPC. HFPG was defined as FPG >5.4 mmol/L, based on the GBD 2021 definition of metabolic risk. This range reflects the threshold above which the risk of EOPC begins to increase, even below the diabetes cut-off (28). For comparison, diabetes is defined as FPG ≥7.0 mmol/L (126 mg/dL). Detailed methodologies have been described previously (25, 29). HBMI was defined as BMI > 25 kg/m^2^ in the GBD 2021. The risk factor information for EOPC in GBD 2021 can be accessed at https://ghdx.healthdata.org/record/ihme-data/gbd-2021-relative-risks.

Within the GBD 2021 framework, the SDI serves as a composite measure of societal development levels, scaled from 0 (lowest) to 1 (highest), and is designed to evaluate regional proximity and similarities in epidemiological patterns. Based on the SDI criteria, 204 nations and territories were stratified into five socioeconomic categories and 21 epidemiological regions. Population statistics derived from the GBD 2021 database were used to model the health effects of EOPC associated with HFPG and HBMI.

### 2.3 Data and statistical analysis

To analyze the change trends of age-standardized rates for EOPC attributable to HFPG and HBMI between 1990 and 2021, the average annual percentage change (AAPC) and corresponding 95% confidence intervals (CI) were derived using the Joinpoint regression analysis tool (Version 5.0). This method constructs a piecewise regression model based on the time characteristics of the disease distribution and then divides the study period into discrete intervals. Each interval undergoes systematic trend fitting and optimization to quantify the temporal patterns of disease variation across global regions (30).

The restricted cubic spline is a widely used method for analyzing nonlinear relationships. This method relies on the spline function principle, dividing the independent variable into several segments within each segment to construct a low-order polynomial function and ensure continuity and smoothness at the knots and first- and second-derivative continuous points, thereby accurately capturing the complex nonlinear relationships (31). The smoothness of the model enhances the precision of depicting variations in survival risk over time or in conjunction with other factors, thereby boosting its predictive accuracy for survival. This involves piecewise polynomial regression, which ensures continuity and second-order differentiability at each node, to produce a smooth curve. The spline function is linear within the range of the independent variable, with the intervals at both ends serving as reference points. The selection of suitable function nodes usually ranges from three to five based on the size of the sample. In this study, three nodes were used as recommended for optimal results (32).

Quantile regression: This method regresses the conditional quantile of a dependent variable on an independent variable, thereby generating a regression model for all the quantiles. It is based on the weighted least-squares method and minimizes the weighted absolute deviation. Quantile regression is particularly useful for non-normally distributed data and provides more comprehensive insights than traditional regression (33). The objective function for quantile regression is:


$$\min\{w_{t}|y_{t}-\alpha =-\sum_{i:\;y_{i}<\alpha }^{T}(1-\tau )(y_{t}-\alpha )+\sum_{i:\;y_{i}\geq \alpha }^{T}\tau (y_{t}-\alpha )$$

Bayesian age-period-cohort (BAPC) analysis: This model utilizes a second-order random walk model to even out initial mortality rates associated with age, time period, and cohort influences, and applies the Integrated Nested Laplace Approximation technique to estimate marginal posterior distributions (34). It combines sample and prior information to produce robust parameter estimates. The BAPC package (R version 4.3.2) was employed to predict incidence trends from 2022 to 2030 to inform public health policies and prevention strategies for EOPC. The statistical significance threshold was set at *P* < 0.05.

## 3 Results

### 3.1 Summary of ASMR and ASDR for EOPC attributable to HFPG (15–49 years, 1990-2021), categorized by regional classifications

**Figure 1:**
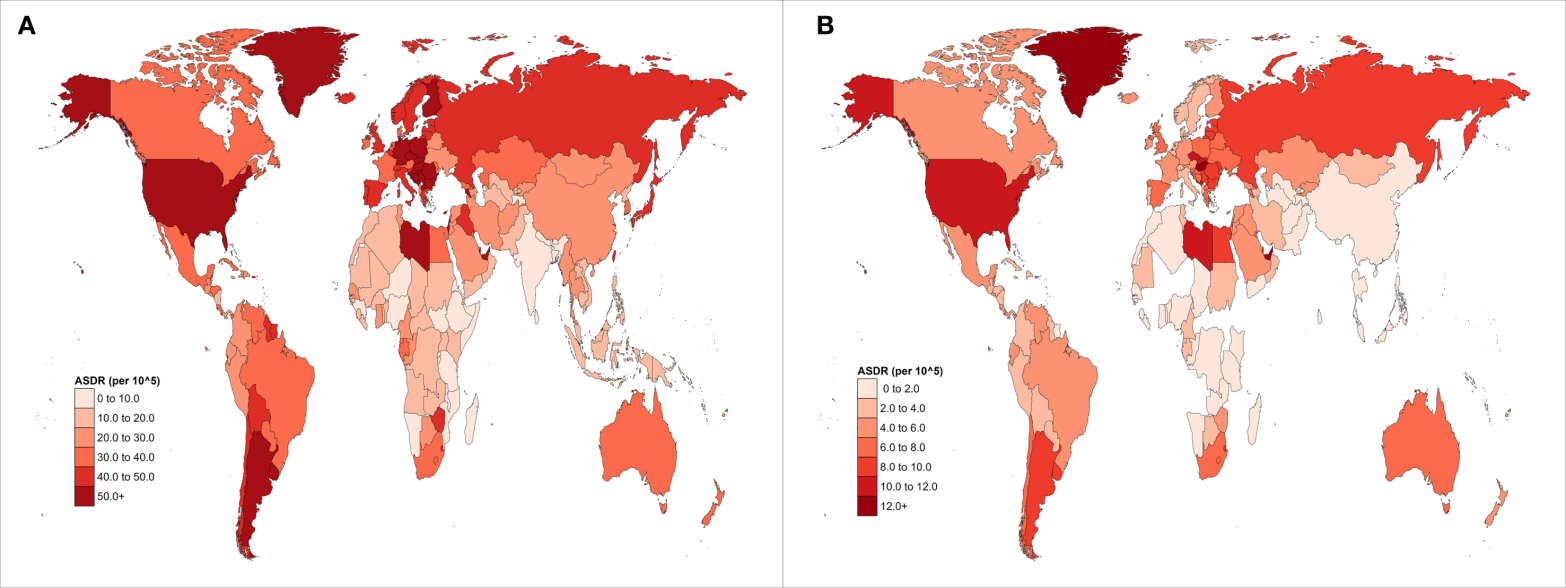
Global distribution maps of ASDR from EOPC attributable to **(A)** HFPG and **(B)** HBMI from 1990 to 2021.

In 2021, EOPC attributed to HFPG resulted in 3,334 global deaths, representing a 2.3-fold increase compared with 1990. Globally, the ASMR and ASDR for HFPG-related EOPC were 0.08, 95% CI = [0.01, 0.16] and 3.91, 95% CI = [0.45, 7.47] per 100,000 people, respectively. From 1990 to 2021, the AAPCs for ASDR and ASMR were 1.47% (95% CI = [1.26, 1.69]) and 1.50% (95% CI = [1.28, 1.72]), respectively (all *P* < 0.001). Across the SDI quintiles, the most substantial increase occurred in the low-middle SDI category, with AAPC values of 2.86% (95% CI = [2.63, 3.08]) for ASMR and 2.84% (95% CI = [2.62, 3.06]) for ASDR (all *P* < 0.001). Regionally, Central Asia exhibited the highest annual growth rates for both ASMR (AAPC = 3.51%, 95% CI = [2.61, 4.43]) and ASDR (AAPC = 3.40%, 95% CI = [2.19, 4.62]) (all *P* < 0.001) (**Table 1**). Notably, East Asia had the highest ASDR for EOPC, attributed to HFPG globally, at 8.29 (95% CI = [0.93, 16.45]) per 100,000 population (**Figure 1A**).

**Table 1:** Summary of age-standardized mortality rates (ASMR) and age-standardized disability rates (ASDR) temporal progression related to HFPG of early-onset pancreatic cancer aged 15–49 years from 1990 to 2021, Stratified by Region.

Location	ASMR in 2021 (per 100,000)	AAPC (95% CI)	*P* value	ASDR in 2021 (per 100,000)	AAPC (95% CI)	*P*-value
Global	0.08 (0.01 to 0.16)	1.50 (1.28 to 1.72)	< 0.001	3.91 (0.45 to 7.47)	1.47 (1.26 to 1.69)	< 0.001
SDI						
High SDI	0.15 (0.02 to 0.28)	1.32 (1.17 to 1.46)	< 0.001	6.83 (0.79 to 12.69)	1.30 (1.17 to 1.43)	< 0.001
High-middle SDI	0.17 (0.02 to 0.32)	2.21 (1.87 to 2.55)	< 0.001	7.77 (0.89 to 15.01)	2.16 (1.80 to 2.52)	< 0.001
Middle SDI	0.09 (0.01 to 0.17)	2.32 (2.14 to 2.49)	< 0.001	4.04 (0.46 to 7.75)	2.22 (2.05 to 2.39)	< 0.001
Low-middle SDI	0.03 (0.00 to 0.07)	2.86 (2.63 to 3.08)	< 0.001	1.59 (0.19 to 3.15)	2.84 (2.62 to 3.06)	< 0.001
Low SDI	0.02 (0.00 to 0.03)	1.32 (1.14 to 1.50)	< 0.001	0.76 (0.09 to 1.51)	1.32 (1.15 to 1.50)	< 0.001
Region						
Andean Latin America	0.05 (0.01 to 0.11)	2.40 (1.75 to 3.06)	< 0.001	2.39 (0.27 to 5.11)	2.38 (1.72 to 3.04)	< 0.001
Australasia	0.12 (0.01 to 0.22)	2.37 (1.90 to 2.83)	< 0.001	5.50 (0.61 to 10.14)	2.34 (1.88 to 2.80)	< 0.001
Caribbean	0.11 (0.01 to 0.22)	2.98 (2.61 to 3.35)	< 0.001	5.10 (0.60 to 10.15)	2.94 (2.60 to 3.28)	< 0.001
Central Asia	0.07 (0.01 to 0.14)	3.51 (2.61to 4.43)	< 0.001	3.03 (0.33 to 6.26)	3.40 (2.19 to 4.62)	< 0.001
Central Europe	0.19 (0.02 to 0.36)	1.33 (1.07 to 1.59)	< 0.001	8.65 (0.99 to 16.25)	1.25 (0.99 to 1.52)	< 0.001
Central Latin America	0.11 (0.01 to 0.20)	1.85 (1.27 to 2.44)	< 0.001	4.88 (0.63 to 9.22)	1.81 (1.24 to 2.39)	< 0.001
Central Sub-Saharan Africa	0.03 (0.00 to 0.07)	0.72 (0.50 to 0.93)	< 0.001	1.46 (0.17 to 3.09)	0.77 (0.64 to 0.89)	< 0.001
East Asia	0.18 (0.02 to 0.35)	2.79 (2.47 to 3.12)	< 0.001	8.29 (0.93 to 16.45)	2.74 (2.47 to 3.01)	< 0.001
Eastern Europe	0.16 (0.02 to 0.31)	2.01 (1.48 to 2.54)	< 0.001	7.39 (0.76 to 14.38)	1.96 (1.44to 2.49)	< 0.001
Eastern Sub-Saharan Africa	0.01 (0.00 to 0.03)	1.76 (1.65 to 1.86)	< 0.001	0.56 (0.06 to 1.18)	1.77 (1.66 to 1.88)	< 0.001
High-income Asia Pacific	0.14 (0.02 to 0.27)	0.51 (0.14 to 0.87)	0.006	6.34 (0.70 to 12.16)	0.45 (0.10 to 0.80)	0.012
High-income North America	0.17 (0.02 to 0.31)	1.70 (1.38 to 2.01)	< 0.001	7.80 (0.96 to 14.10)	1.68 (1.37 to 2.00)	< 0.001
North Africa and Middle East	0.08 (0.01 to 0.15)	3.43 (3.34 to 3.52)	< 0.001	3.68 (0.44 to 7.05)	3.41 (3.32 to 3.49)	< 0.001
Oceania	0.05 (0.01 to 0.10)	1.48 (1.36 to 1.59)	< 0.001	2.36 (0.27 to 4.82)	1.51 (1.40to 1.62)	< 0.001
South Asia	0.03 (0.00 to 0.05)	2.31 (2.01 to 2.62)	< 0.001	1.26 (-0.16 to 2.39)	2.30 (2.06 to 2.55)	< 0.001
Southeast Asia	0.05 (0.01 to 0.10)	3.12 (2.87 to 3.37)	< 0.001	2.34 (0.27 to 4.79)	3.06 (2.81 to 3.31)	< 0.001
Southern Latin America	0.12 (0.01 to 0.23)	1.59 (1.11 to 2.07)	< 0.001	5.34 (0.61 to 10.40)	1.60 (1.12 to 2.09)	< 0.001
Southern Sub-Saharan Africa	0.06 (0.01 to 0.13)	2.25 (1.91 to 2.58)	< 0.001	2.97 (0.30 to 6.04)	2.20 (1.84to 2.56)	< 0.001
Tropical Latin America	0.12 (0.01 to 0.22)	2.68 (2.35 to 3.01)	< 0.001	5.47 (0.65 to 10.32)	2.64 (2.32 to 2.96)	< 0.001
Western Europe	0.11 (0.01 to 0.21)	1.04 (0.86 to 1.23)	< 0.001	5.00 (0.54 to 9.75)	1.04 (0.85 to 1.24)	< 0.001
Western Sub-Saharan Africa	0.02 (0.00 to 0.03)	3.03 (2.94 to 3.12)	< 0.001	0.72 (0.08 to 1.44)	3.05 (2.96 to 3.14)	< 0.001

### 3.2 Summary of ASMR and ASDR and temporal progression related to HBMI of EOPC aged 15–49 years from 1990 to 2021, stratified by region

Globally, the ASMR and ASDR for EOPC attributable to HBMI were 0.01, 95% CI = [0.00, 0.03] and 0.54, 95% CI = [-0.16, 1.62] per 100,000 population in 2021. From 1990 to 2021, the AAPC for ASMR and ASDR was 1.69%, 95% CI = [1.62%, 1.77%] and 1.76%, 95% CI = [1.69%, 1.84%], respectively (all *P* values< 0.001). Among SDI categories, Low-middle SDI areas showed the highest rate of increase, with AAPC values of 2.86%, 95% CI = [2.63, 3.08] for ASMR and 2.84%, 95% CI = [2.62, 3.06] for ASDR (all *P* values< 0.001). Regionally, Central Asia exhibited the highest annual growth rates for both ASMR (AAPC = 3.51%, 95% CI = [2.61, 4.43]) and ASDR (AAPC = 3.40%, 95% CI = [2.19, 4.62%]), all *P* values< 0.001 (**Table 2**). Moreover, High-Income North America had the highest ASDR for EOPC attributed to HBMI globally, at 2.45, 95% CI = [0.06, 5.35] per 100,000 population (**Figure 1B**).

**Table 2:** Summary of age-standardized mortality rates (ASMR) and age-standardized disability rates (ASDR) and temporal progression related to HBMI of early-onset pancreatic cancer aged 15–49 years from 1990 to 2021, Stratified by Region.

Location	ASMR in 2021 (per 100,000)	AAPC (95% CI)	*P* value	ASDR in 2021 (per 100,000)	AAPC (95% CI)	*P* value
Global	0.01 (-0.00 to 0.03)	1.69 (1.62 to 1.77)	< 0.001	0.54 (-0.16 to 1.62)	1.76 (1.69 to 1.84)	< 0.001
SDI						
High SDI	0.03 (-0.00 to 0.08)	4.75 (4.32 to 5.18)	< 0.001	1.44 (-0.08 to 3.61)	4.86 (4.39 to 5.34)	< 0.001
High-middle SDI	0.02 (-0.01 to 0.07)	3.02 (2.78 to 3.25)	< 0.001	1.08 (-0.37 to 3.34)	3.16 (2.92 to 3.40)	< 0.001
Middle SDI	0.01 (-0.00 to 0.03)	2.02 (1.96 to 2.07)	< 0.001	0.42 (-0.20 to 1.42)	2.12 (2.06 to 2.18)	< 0.001
Low-middle SDI	0.00 (-0.00 to 0.01)	0.79 (0.76 to 0.81)	< 0.001	0.20 (-0.07 to 0.62)	0.81 (0.79 to 0.84)	< 0.001
Low SDI	0.00 (-0.00 to 0.00)	0.28 (0.27 to 0.29)	< 0.001	0.01 (-0.09 to 0.16)	0.28 (0.27 to 0.29)	< 0.001
Region						
Andean Latin America	0.02 (-0.00 to 0.05)	6.67 (6.07 to 7.27)	< 0.001	0.95 (-0.05 to 2.54)	7.01 (6.39 to 7.63)	< 0.001
Australasia	0.04 (-0.00 to 0.09)	4.00 (3.23 to 4.78)	< 0.001	1.69 (-0.03 to 4.09)	4.11 (3.20 to 5.02)	< 0.001
Caribbean	0.02 (-0.00 to 0.06)	6.45 (5.90 to 6.97)	< 0.001	1.14 (-0.08 to 3.02)	6.48 (5.94 to 7.02)	< 0.001
Central Asia	0.02 (-0.00 to 0.05)	5.48 (4.39 to 6.58)	< 0.001	0.71 (-0.16 to 2.29)	6.00 (4.94 to 7.07)	< 0.001
Central Europe	0.04 (-0.00 to 0.11)	2.29 (1.85 to 2.74)	< 0.001	1.90 (-0.17 to 5.02)	2.32 (1.86 to 2.78)	< 0.001
Central Latin America	0.03 (-0.00 to 0.07)	4.75 (4.25 to 5.25)	< 0.001	1.36 (-0.03 to 3.25)	4.84 (4.35 to 5.34)	< 0.001
Central Sub-Saharan Africa	0.00 (-0.00 to 0.01)	0.67 (0.65 to 0.68)	< 0.001	0.07 (-0.10 to 0.35)	0.68 (0.66 to 0.70)	< 0.001
East Asia	0.01 (-0.00 to 0.04)	5.04 (4.70 to 5.38)	< 0.001	0.23 (-0.69 to 1.88)	5.73 (5.34 to 6.13)	< 0.001
Eastern Europe	0.05 (-0.00 to 0.13)	4.29 (3.58 to 5.01)	< 0.001	2.37 (-0.23 to 6.23)	4.37 (3.65 to 5.10)	< 0.001
Eastern Sub-Saharan Africa	0.00 (-0.00 to 0.00)	0.37 (0.36 to 0.38)	< 0.001	0.02 (-0.10 to 0.21)	0.39 (0.38 to 0.40)	< 0.001
High-income Asia Pacific	-0.01 (-0.02 to 0.02)	8.59 (6.25 to 10.97)	< 0.001	-0.29 (-0.87 to 0.69)	9.99 (7.26 to 12.80)	< 0.001
High-income North America	0.05 (0.00 to 0.12)	2.18 (1.76 to 2.60)	< 0.001	2.45 (0.06 to 5.35)	2.17 (1.84 to 2.51)	< 0.001
North Africa and Middle East	0.03 (0.00 to 0.06)	5.90 (5.75 to 6.06)	< 0.001	1.27 (0.02 to 2.89)	5.99 (5.83 to 6.16)	< 0.001
Oceania	0.01 (-0.00 to 0.02)	3.85 (3.59 to 4.11)	< 0.001	0.36 (-0.04 to 1.01)	3.89 (3.61 to 4.17)	< 0.001
South Asia	0.00 (-0.00 to 0.00)	0.24 (0.23 to 0.25)	< 0.001	0.01 (-0.08 to 0.17)	0.24 (0.23 to 0.25)	< 0.001
Southeast Asia	0.00 (-0.00 to 0.01)	0.78 (0.77 to 0.80)	< 0.001	0.06 (-0.30 to 0.67)	0.83 (0.81 to 0.84)	< 0.001
Southern Latin America	0.04 (-0.00 to 0.09)	3.22(2.74 to 3.71)	< 0.001	1.76 (-0.07 to 4.40)	3.42 (2.97 to 3.87)	< 0.001
Southern Sub-Saharan Africa	0.03 (-0.00 to 0.06)	4.21 (3.48 to 4.96)	< 0.001	1.18 (-0.04 to 2.98)	4.31 (3.56 to 5.07)	< 0.001
Tropical Latin America	0.03 (-0.00 to 0.07)	6.30 (5.86 to 6.75)	< 0.001	1.35 (-0.09 to 3.48)	6.57 (6.15 to 6.99)	< 0.001
Western Europe	0.02 (-0.00 to 0.07)	4.56 (4.33 to 4.79)	< 0.001	1.07 (-0.20 to 3.06)	4.73 (4.48 to 4.98)	< 0.001
Western Sub-Saharan Africa	0.00 (-0.00 to 0.01)	0.36 (0.35 to 0.37)	< 0.001	0.08 (-0.06 to 0.32)	0.37 (0.36 to 0.38)	< 0.001

### 3.3 Summary of ASMR and ASDR for EOPC attributable to high FPG (15–49 years, 1990-2021), categorized by SDI classifications

**Figure 2:**
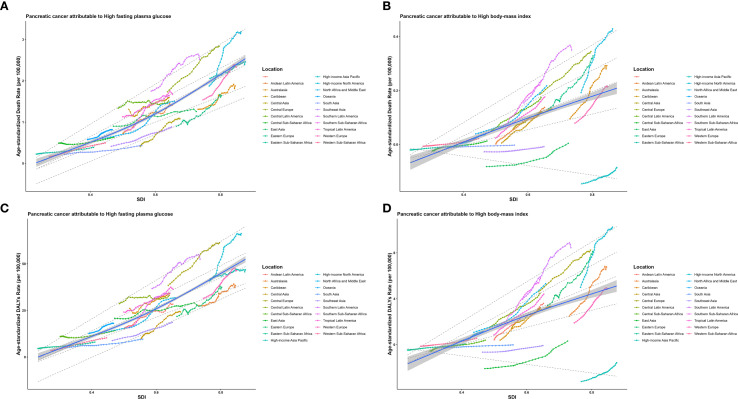
The relationships between the ASMR and ASDR of EOPC caused by HFPG, HBMI and SDI worldwide and in 21 regions from 1990 to 2021. The middle blue line is the fitted regression line for the SDI - disease burden relationship (representing the average trend), and the surrounding shaded areas represent 95% CI. **(A, B)** illustrate the correlation between ASMR of EOPC attributable to HFPG/HBMI and SDI, while **(C, D)** show the correlation between ASDR of EOPC attributable to HFPG/HBMI and SDI.

Based on the GBD classification, this study collected ASMR, YLLs, and SDI data from 21 different regions. By applying RCS and quantile regression, the study explored the correlation between these indicators and SDI. As illustrated in **Figure 2A**, the ASMR of EOPC attributable to HFPG exhibited a significant positive correlation with SDI. Quantile regression analysis revealed that at the lowest quantile (5th percentile), SDI was negatively correlated with ASMR. At the 25th, 50th, 75th, and 90th percentiles, SDI was positively correlated with ASMR. The correlation between the ASMR of EOPC attributable to HBMI and SDI was consistent with that of HFPG (**Figure 2B**). The relationship between ASDR and SDI also yielded the same results (**Figures 2C, D**).

### 3.4 Projections of mortality to 2030 for EOPC attributable to HFPG and HBMI

**Figure 3:**
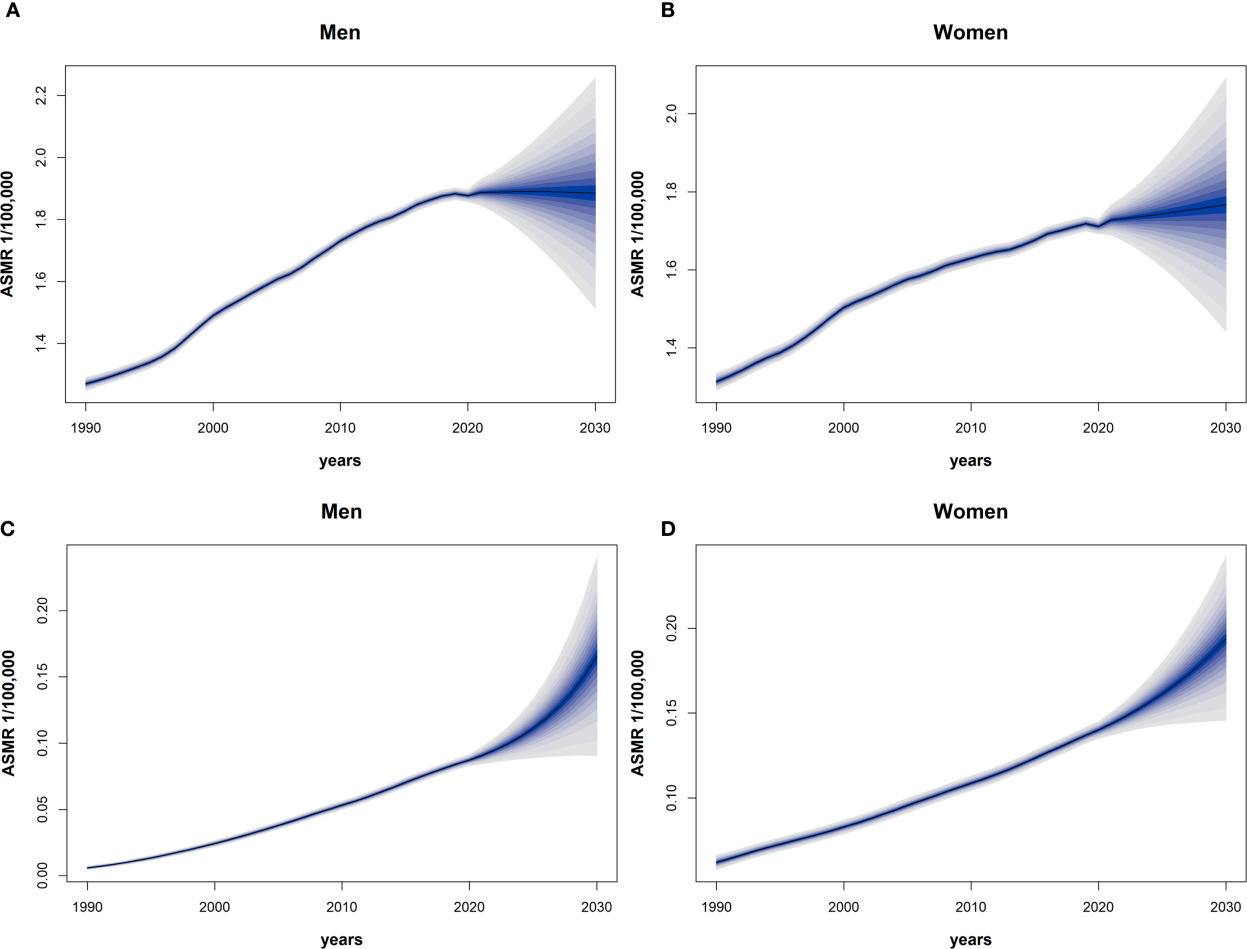
The 1990–2030 global time trend of ASMR for EOPC from HFPG and HBMI, by sex. Dark blue solid lines are predicted ASMR trends; light blue shaded areas represent 95% CI. **(A–D)** Predictions for males [**(A)** HFPG, **(C)** HBMI] and females [**(B)** HFPG, **(D)** HBMI].

Based on the Bayesian model, we predicted the ASMR of EOPC attributable to HFPG and HBMI from 2021 to 2030. The results revealed that in males, the ASMR of EOPC due to HFPG would slightly decrease from 18.87 to 18.85, whereas in females, it would increase from 17.27 to 17.66. For EOPC attributable to HBMI, the ASMR in males would rise from 0.90 to 1.65, and in females, it would increase from 1.43 to 1.93. Over the next decade, the ASMR of EOPC due to HFPG in males would remain higher than that in females (**Figures 3A, B**), while ASMR attributable to HBMI would be higher in females than in males (**Figures 3C, D**). Overall, these findings indicate that the mortality burden of EOPC attributable to HFPG and HBMI is on an upward trend over the next decade.

## 4 Discussion

This study provides a comprehensive analysis and projection of the global disease burden of early-onset pancreatic cancer (EOPC) attributable to high fasting plasma glucose (HFPG) and high body mass index (HBMI). The results demonstrate distinct geographical and temporal variations in the impact of these metabolic risk factors on EOPC: globally, the annual increases in the ASMR of EOPC linked to HFPG and HBMI were 1.50% and 2.16%, respectively, with the most significant increases observed in low- and middle-SDI regions (ASMR growth rates reaching 2.86% and 3.12%, respectively). Regionally, East Asia, North America, and Western Europe bear the highest absolute burden of such EOPC, while Central Asia and Southeast Asia show the fastest growth rates. Predictions for 2030 indicate that EOPC mortality associated with these metabolic factors will continue to rise—particularly among women, where the impact of HBMI may exceed that of HFPG. The study emphasizes the critical role of metabolic risk factors in EOPC, and recommends enhanced control of obesity and diabetes to reduce EOPC cases linked to these factors.

Our analysis reveals striking regional disparities in the burden of early-onset pancreatic cancer. While high-income countries carry the highest absolute burden, the most rapid increases are occurring in low- to middle-SDI regions. Taking HFPG as an example, the ASMR in high-income North America is 8.5 times that of sub-Saharan Africa, yet the latter’s average annual growth rate from 1990 to 2021 (2.86%) far exceeds that of the former (1.70%). This “dual burden” mirrors shifting global exposure: longstanding high-calorie diets and sedentary lifestyles keep obesity and diabetes high in affluent countries (35); whereas rapid urbanization is reshaping lifestyles in low-income countries—Southeast Asia is seeing an increase in unhealthy diets and a decrease in physical activity (36), while sub-Saharan Africa has experienced a significant rise in the consumption of sugary beverages (37).Critically, unequal healthcare capacity magnifies these gaps: comprehensive screening and cancer control programs in high-SDI countries improve early detection and survival (38). Yet weak infrastructure in low-SDI regions delays diagnosis until advanced stages, yielding elevated mortality-to-incidence ratio (39). Quantile analyses further reveal a nonlinear SDI–ASMR relationship: at lower quantiles, SDI is negatively associated with ASMR, possibly reflecting underdiagnosis or data gaps in the poorest areas (40); at medium-to-high quantiles, SDI correlates positively, which may be related to economic development driving the westernization of lifestyles, which in turn increases metabolic risks (41–43).

This study also underscores the significance of gender differences in the disease burden of metabolic-related EOPC. Findings show that the ASMR and ASDR of EOPC are generally higher in men than in women, with a male-to-female ASMR ratio of 1.19:0.67; these differences are more pronounced particularly in low- and middle-SDI regions. This phenomenon may stem from multiple factors. First, men are more frequently exposed to co-risk factors such as smoking and alcohol consumption. Globally, male smoking prevalence is approximately 34% compared to 6% in women (44), and research has confirmed that smoking significantly increases the mortality risk of patients with pancreatic cancer, with male smoking prevalence generally higher than that in females (45). Second, hormonal differences may affect the impact of metabolic risk factors—for instance, androgens can enhance obesity-related carcinogenic effects by regulating fat distribution and inflammatory responses (46, 47). Additionally, gender differences in social roles may lead to lower health awareness among men, resulting in more common delays in seeking medical care, which in turn exacerbates prognostic disparities. Furthermore, the study’s predictive model indicates that by 2030, HBMI-related EOPC ASMR will continue to rise in both genders. In contrast, HFPG-related burden will show a slight decline in men but persistently rise in women. This result suggests that future interventions targeting metabolic risk factors need to pay more attention to gender differences, especially in the management of obesity and diabetes in women.

The changing behavior patterns of the youth population may be a significant driver of the rising burden of EOPC. Globally, the obesity rate among adolescents has significantly increased over the past 30 years (48), the global prevalence of obesity in adolescents increased from 0.7% in 1975 to 5.6% in 2016 in girls, and from 0.9% to 7.8% in boys (49), and the age at which diabetes manifests in the youth population is exhibiting a trend towards earlier onset (50). Additionally, co-occurring risk factors, including tobacco use and alcohol consumption, are increasingly prevalent among the youth in low- and middle-income nations. For example, the smoking rate among young men in Southeast Asia is to the extent of 40% (51), which, combined with HFPG and HBMI, further exacerbates the risk of PC. The interaction of these behavioral factors with metabolic abnormalities may explain why the incidence of EOPC in Asian men is increasing significantly faster than in other regions. Furthermore, molecular profiling suggests that EOPC patients exhibit distinct characteristics compared to older cohorts, including a higher prevalence of CDKN2A deletions and a lower frequency of SMAD4 mutations (52, 53), This implies that metabolic stress may induce specific pathway dysregulations via epigenetic mechanisms (54). However, these mechanisms remain hypothetical and require further validation through dedicated molecular studies. These findings emphasize the necessity of conducting metabolic interventions targeting the youth population.

The advantage of this study lies in its first-time use of the GBD big data platform to reveal the impact of metabolic risks on global EOPC and its application of a Bayesian age-period-cohort model for dynamic predictions, providing spatiotemporal evidence support for global prevention and control. However, the following limitations should be noted: First, it relies on GBD 2021 methods, failing to adjust for known but unmodeled risk factors (e.g., smoking, alcohol), which may inflate metabolic-attributable fractions and mask synergistic/antagonistic effects; Second, it lacks stratified analyses (e.g., age, ethnicity, healthcare access), preventing the identification of high-risk subpopulations and reducing granularity for precision public health planning; Third, scarce high-quality cancer registry data in low/middle-SDI regions may underestimate true EOPC incidence/mortality, weakening observed growth rates and misleading EOPC control priority-setting; Fourth, the projections were not subjected to formal cross-validation or explicit uncertainty quantification beyond the 95% uncertainty intervals already provided by the GBD 2021 DisMod-MR 2.1 framework; consequently, the robustness of the forecasted trends remains to be externally validated. Future work should prioritize four key dimensions: incorporating stratified analyses (e.g., by age subgroups, regional characteristics) to identify populations most heavily affected by metabolic-related EOPC; improving data quality to correct the underestimated disease burden and thereby clarify its implications for study conclusions; including confounding variables to explore causal associations between metabolic risks and EOPC.

## 5 Conclusion

This study comprehensively reveals for the first time the disease burden of EOPC attributed to metabolic risks among the global population aged 15-49, along with its spatiotemporal evolution. The results indicate that the burden of EOPC related to metabolic risks remains high in high-SDI regions, while it is experiencing rapid growth in middle- and low-income regions. This trend is closely related to the global epidemics of obesity and diabetes, unequal distribution of medical resources, and changes in youth behavior patterns. Implementing integrated policy, educational, and healthcare strategies—particularly by enhancing the prevention and management of metabolic disorders in low- and middle-SDI regions—may help curb the rising incidence of pancreatic cancer. In the future, multidisciplinary collaboration and precise intervention strategies are needed to further reduce the threat of this aggressive cancer to global health.

## Data Availability

The original contributions presented in the study are included in the article/supplementary material. Further inquiries can be directed to the corresponding author.
